# PBMCpedia: a harmonized PBMC scRNA-seq database with unified mapping and enhanced celltype annotation

**DOI:** 10.1093/nar/gkaf1245

**Published:** 2025-11-24

**Authors:** Emma S Hoffmann, Lara Hombrecher, Ian F Diks, Matthias Flotho, Pascal Hirsch, Andreas Keller, Friederike Grandke

**Affiliations:** Clinical Bioinformatics, Saarland University, 66123 Saarbrücken, Germany; Clinical Bioinformatics, Saarland University, 66123 Saarbrücken, Germany; Clinical Bioinformatics, Saarland University, 66123 Saarbrücken, Germany; Clinical Bioinformatics, Saarland University, 66123 Saarbrücken, Germany; Helmholtz Institute for Pharmaceutical Research Saarland–Helmholtz Centre for Infection Research, 66123 Saarbrücken, Germany; Clinical Bioinformatics, Saarland University, 66123 Saarbrücken, Germany; Clinical Bioinformatics, Saarland University, 66123 Saarbrücken, Germany; Helmholtz Institute for Pharmaceutical Research Saarland–Helmholtz Centre for Infection Research, 66123 Saarbrücken, Germany; PharmaScienceHub, Saarland University, 66123 Saarbrücken, Germany; Clinical Bioinformatics, Saarland University, 66123 Saarbrücken, Germany; Helmholtz Institute for Pharmaceutical Research Saarland–Helmholtz Centre for Infection Research, 66123 Saarbrücken, Germany

## Abstract

Single-cell transcriptomic studies of peripheral blood mononuclear cells (PBMCs) offer valuable insights into immune states across diverse biological conditions, yet cross-study integration remains difficult due to divergent preprocessing and annotations. PBMCpedia addresses this by uniformly reprocessing 519 samples (over 4.3 million cells) from 24 publicly available single-cell RNA sequencing studies using a standardized pipeline with consistent quality control and hierarchical cell type annotation. Spanning 14 disease contexts, including autoimmune, infectious, and neurodegenerative disorders, as well as healthy controls, PBMCpedia supports metadata-aware comparisons across diseases, cell types, sexes, and age groups. It also includes T-cell receptor/B-cell receptor repertoire data for 75 samples and surface protein measurements for 56 samples, enabling integrative immune profiling at both the transcriptomic and proteogenomic levels. To support exploration and accessibility, we provide an interactive web interface (https://web.ccb.uni-saarland.de/pbmcpedia/) for querying gene expression, marker genes, and pathway enrichment across cell types, conditions, sexes, and age groups. PBMCpedia fills a critical gap by offering a transparent, harmonized, and disease-diverse PBMC resource designed for cross-study immune profiling and discovery.

## Graphical Abstract

**Graphical Abstract:**
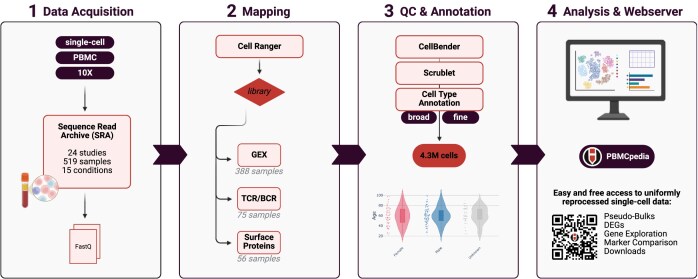

## Introduction

Single-cell RNA sequencing (scRNA-seq) of peripheral blood mononuclear cells (PBMCs) is a powerful approach to profile immune heterogeneity across diverse biological contexts, including infection, autoimmunity, aging, and neurodegeneration. Despite the availability of hundreds of public PBMC datasets, comparative analyses remain difficult due to inconsistent preprocessing, divergent annotation schemes, and batch effects. These challenges limit reproducibility and hinder the reuse of existing datasets.

Several large-scale PBMC atlases have addressed specific aspects of this issue. The Allen Institute’s Human Immune Health Atlas [1] includes over 16 million immune cells from healthy donors and provides a standardized annotation framework stratified by age, but excludes disease cohorts. Jiménez-Gracia *et al.* [2] reprocessed raw data from 356 patients across 18 inflammatory conditions, generating the “Inflammation Landscape” using interpretable machine learning. However, their harmonized dataset is not accessible via an interactive platform or downloadable in full. Connolly *et al.* [3] created a PBMC aging atlas by integrating 2.8 million cells from 35 studies, but used heterogeneous preprocessing pipelines and do not provide a centralized portal. CancerSCEM 2.0 [4] aggregates over 1400 datasets across 74 cancer types, with limited emphasis on PBMCs.

To address these limitations, we developed PBMCpedia, a harmonized, multidisease PBMC atlas comprising 4 293 193 single-cell transcriptomes from 519 samples across 24 publicly available scRNA-seq studies (Supplementary Table S1). PBMCpedia introduces three core advances. First, all FASTQ files are uniformly reprocessed with standardized quality control, batch correction, and hierarchical annotation, ensuring comparability across studies. Whenever available, all samples include metadata on age, sex, and disease status, enabling stratified, demographic-aware analyses. Second, the resource integrates downstream analysis modules—including differential expression and pathway enrichment—exposed through a reproducible, application programming interface (API)-backed web interface. Third, PBMCpedia combines transcriptomic profiles with paired T-cell receptor (TCR)/B-cell receptor (BCR) repertoires and surface protein measurements, enabling multimodal exploration of immune states at single-cell resolution. Together, these features establish PBMCpedia as both a harmonized reference and a practical diagnostic toolkit for cross-study immune analysis, extending beyond prior PBMC atlases by combining breadth, standardization, and accessible downstream functionality.

## Materials and methods

We included 24 publicly available scRNA-seq PBMC datasets covering 14 disease conditions and healthy controls. All datasets were generated with 10x Genomics technology, included raw FASTQ files, and contained usable sample metadata (age, sex, disease status). Datasets lacking raw data or essential metadata were excluded. In total, 519 samples and 4 293 193 high-quality cells were retained following uniform quality control.

### Data acquisition

Sequencing data were retrieved from the Sequence Read Archive (SRA) using the SRA Toolkit (v3.0.0). We downloaded .sra files using prefetch, verified them with vdb-validate, and extracted paired reads using fasterq-dump with –-split-files and –-include-technical to preserve index reads. Because submission formats varied, files were manually curated to ensure correct pairing and orientation.

Metadata were collected via literature review and the NCBI SRA Run Selector. Age was treated as a continuous variable; sex was categorized as male, female, or unknown to reflect inconsistent reporting.

### Uniform preprocessing and quality control

All datasets were aligned with Cell Ranger (v9.0.0) to GRCh38-2024-A. Count matrices were processed using Scanpy (v1.11.1) [5], CellBender (v0.3.2) [6] for ambient RNA correction, and Scrublet (via Scanpy) [7] for doublet detection. Doublet detection was performed on a per-sample basis using a combined feature matrix that included both transcriptomic and surface protein measurements whenever CITE-seq data were available. Cells with fewer than 200 genes or above the 98th percentile for gene/unique molecular identifier (UMI) counts were removed. We did not apply a strict filter based on mitochondrial gene content because, in PBMC datasets, distinguishing truly stressed or low-quality cells from biologically relevant variation in mitochondrial reads is challenging. Immune cell subsets naturally vary in mitochondrial content, and applying a universal cutoff could disproportionately remove specific cell types.

Integration was performed using Harmony (via harmonypy, version 0.0.10) [8] on 15 PCs, correcting batch effects by project and sample ID. UMAP visualizations confirmed dataset mixing and clustering by immune identity. UMAP for webserver was downsampled to 10k cells per cell type.

Multiplexed samples were demultiplexed by mapping cells to published donor assignments. This ensured accurate donor attribution.

### Annotation strategy

Cell type labels were transferred using the Allen Institute’s cell_type_mapper tool at two hierarchical levels: AIFI_L1 (major lineages) and AIFI_L2 (subtypes) using the Human Immune Health Atlas [1] as reference. This annotation supports flexible downstream analysis. The cell type labels were manually verified using known marker genes.

### Differential expression and pathway analysis

Pseudobulk differential gene expression (DGE) analysis was conducted using Scanpy (v1.11.2) [5] and limma (v3.58.1 via rpy2 v3.5.11) [9], comparing disease versus control samples across cell types, annotation levels, sexes, and age groups. We performed preranked GSEA using GSEApy (v1.1.9) with the “GO_Biological_Process_2021” [10, 11] library. Genes were ranked by $\mathrm{ log}_2$ fold-change (logFC), retaining the top 800 by absolute logFC and excluding those with $\mathrm{ |logFC|} < 0.15$. Gene sets with 10–2000 members were considered, using 250 permutations and 3 threads. For each comparison, DGE was computed using the Wilcoxon rank-sum test with Benjamini–Hochberg correction. Stratifications included sex (female/male versus same-sex controls) and age group (young: <25, adult: $25{\hbox {-}}64$, elderly: ${\hbox{>}} 64$), with an additional pooled comparison to maximize power.

### TCR and BCR data extraction

For all datasets containing immune repertoire information, we processed raw FASTQ files using the 10x Genomics Cell Ranger (v9.0.0) vdj or multi pipeline. The corresponding V(D)J reference (GRCh38, 10x Genomics v9.0.0) was used to align reads and assemble full-length, paired TCR and BCR sequences. Output files (filtered_contig_annotations.csv) were parsed to extract chain type, V/D/J gene calls, and pairing information using scirpy (v0.22.1) [12]. Paired $\alpha \beta$ (TCR), and light/heavy (BCR) chain assignments were determined per cell barcode.

### Web server implementation

PBMCpedia is hosted as a Django-based web application with visualizations rendered via Plotly and DataTables. An API provides easy and fast programmatic access to the data. Integrated knowledge bases, including CellMarker [13], PanglaoDB [14], MSigDB [15, 16], and the Human Protein Atlas [17], enhance interpretability. Contextual links for each gene allow real-time reference to known functions and expression profiles.

## Results

### Overview

**Figure 1.**
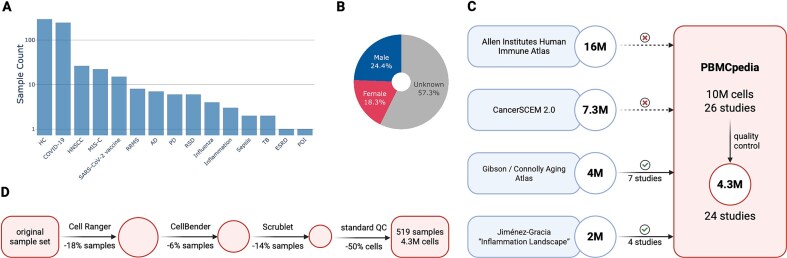
(**A**) Disease distribution. (**B**) Overall sex distribution across all 519 samples. (**C**) Overlap of studies included in PBMCpedia compared to four major PBMC atlases: the Allen Immune Human Health Atlas, Jiménez-Gracia’s Inflammation Landscape, Connolly *et al.*’s aging atlas, and CancerSCEM 2.0. PBMCpedia shares a small subset of studies with Connolly and Jiménez-Gracia but is otherwise largely nonoverlapping. (**D**) Cell and sample filtering across preprocessing steps. Created in BioRender: Hoffmann, E. (2026) https://BioRender.com/lqe6s4z.

PBMCpedia fills a critical need for a harmonized, multidisease PBMC reference by integrating 24 publicly available scRNA-seq datasets into a harmonized reference atlas of human PBMCs. After uniform reprocessing and stringent quality control, the dataset comprises 4 293 193 high-quality transcriptomes from 519 samples, covering 14 disease conditions and healthy controls (Fig. 1A).

The resource spans a broad range of biological contexts, including infectious diseases (e.g. COVID-19, tuberculosis), autoimmune disorders (e.g. systemic lupus erythematosus, rheumatoid arthritis), and neurodegeneration (e.g. Alzheimer’s and Parkinson’s disease). Each sample is (where available) annotated with standardized metadata including disease status, donor sex, and age, enabling stratified analysis across demographic variables (Fig. 1B).

All datasets were processed through a consistent pipeline and integrated using Harmony to correct for batch effects while preserving biological structure. Cells were annotated using a two-level hierarchical framework aligned with the Allen Human Immune Health Atlas, capturing both broad immune lineages and fine-grained subtypes.

Where available, PBMCpedia also includes multimodal profiles: 75 samples contain paired TCR/BCR repertoire sequencing and 56 include CITE-seq-based surface protein quantification. Our final PBMCpedia object retains all three modalities as separate layers. We did not integrate them into a single embedding, leaving users the flexibility to combine or jointly analyze modalities according to their specific research needs.

In total, PBMCpedia provides a reproducible, richly annotated PBMC resource suitable for comparative analysis, biomarker discovery, and immunological exploration across diverse diseases and patient subgroups.

### Comparison to existing resources

Several large-scale PBMC resources exist, but each addresses only part of the challenge. The Allen Human Immune Health Atlas [1] profiles over 16 million immune cells from healthy donors with a focus on age-related changes, but it excludes disease cohorts. Jiménez-Gracia *et al. *[2] reprocessed 2 million PBMCs from 356 patients across 18 inflammatory conditions in the “Inflammation Landscape,” yet the harmonized dataset is not available as a full download or interactive browser. Connolly *et al.* [3] integrated 2.8 million cells from 35 public studies into an aging-focused PBMC atlas, supplemented by a 1.2-million-cell validation cohort. However, they used heterogeneous pipelines and do not provide a unified, interactive portal. CancerSCEM 2.0 [4] aggregates over 1400 datasets across 74 cancer types, including PBMCs. However, its primary emphasis is on tumor microenvironments rather than systemic immune profiling.

PBMCpedia overcomes these limitations by uniformly reprocessing raw FASTQ files using a consistent pipeline with stringet quality control and Harmony-based integration, eliminating batch effects from study-specific workflows. The atlas covers 14 disease contexts and healthy controls, supports hierarchical annotations aligned with the Allen framework, and provides metadata-stratified differential expression and pathway enrichment analyses. Beyond harmonization, PBMCpedia uniquely incorporates multimodal data: 75 samples with paired TCR/BCR repertoires and 56 with CITE-seq surface proteins, enabling joint exploration of transcriptomic, clonotypic, and proteomic variation (Fig. 1 C).

To ensure robust and reproducible insights, we applied strict preprocessing and quality control filters across all studies. These include thresholds for gene and UMI counts, and doublet exclusion based on both transcriptomic and proteomic profiles using Scrublet. While these steps are essential to minimize technical artifacts and ensure biological relevance, they come at a cost: over $50\%$ (5 625 609) of initially captured cells were discarded, resulting in a substantial shrinkage of the dataset. We annotated with a binary flag indicating whether cells exceed a 10% mitochondrial threshold, and the counts per project are reported in Supplementary Table S2, allowing users to assess and optionally filter cells according to their own analytical needs. This rigorous curation underscores PBMCpedia’s focus on data integrity over raw cell quantity, yielding a high-confidence reference atlas suitable for downstream machine learning and systems immunology applications (Fig. 1 D).

All results are freely accessible through a user-friendly web interface, with downloadable data and code to ensure transparency and reuse. PBMCpedia thus fills a critical gap in the single-cell landscape by offering a reproducible, disease-aware resource for comparative immunology.

### Cross-disease immune signatures

**Figure 2.**
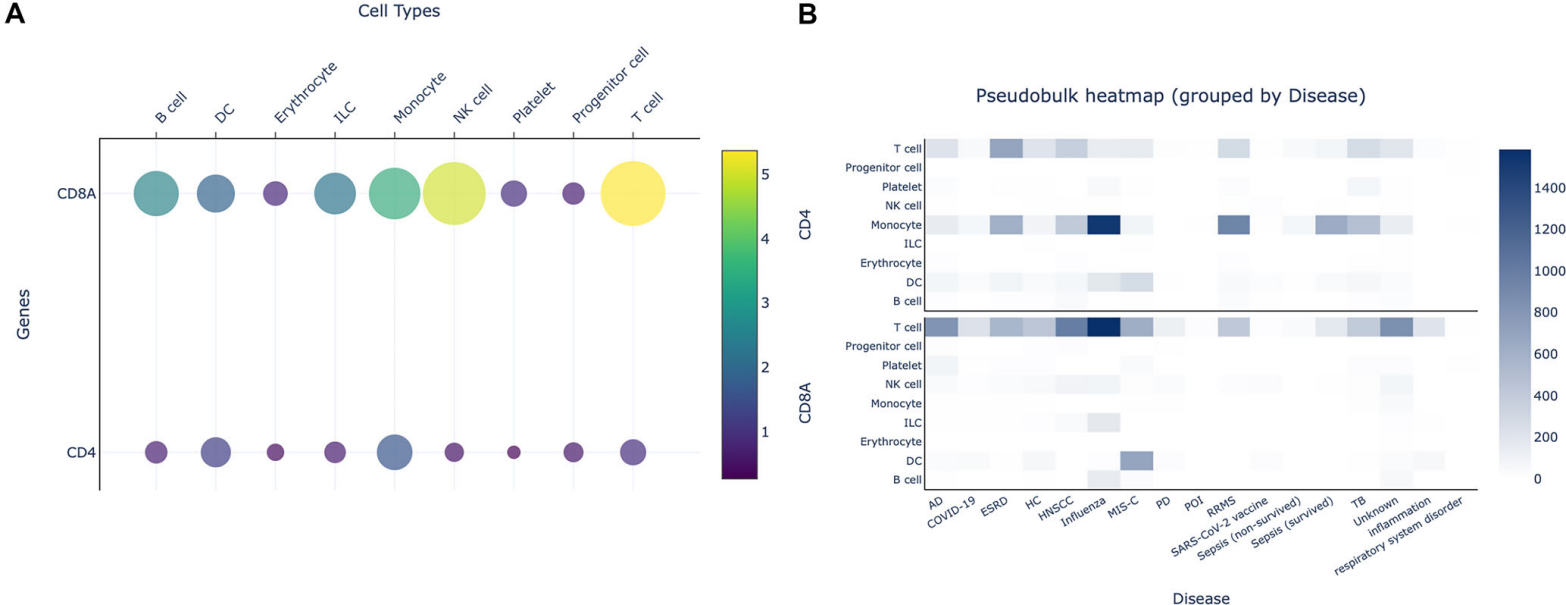
Examples from the PBMCpedia webserver. (**A**) Dot plot of *CD4* and *CD8A*, visualizing their relative expression across annotated cell types at different resolution levels. The dot size represents the proportion of expressing cells, while color intensity reflects average expression. (**B**) Heatmap displaying *CD4* and *CD8A* across broad immune lineages. Created in BioRender: Hoffmann, E. (2026) https://BioRender.com/lqe6s4z.

Because PBMCpedia spans diverse biological contexts, all processed through a unified pipeline, it enables direct, systematic comparisons across conditions (Fig. 2). Users can explore differences and commonalities in immune responses across diseases (e.g. influenza versus COVID-19), age groups (e.g. young versus adult), and sexes (e.g. male versus female) without the confounding effects of batch differences or inconsistent annotations. This level of comparability is difficult to achieve when integrating data from separate studies, where technical variation and divergent labeling schemes often obscure true biological signals.

With that, PBMCpedia provides a framework not only for hypothesis-driven analysis but also for diagnostic exploration, identifying patterns of immune dysregulation that recur across conditions or stratify patient subgroups. This supports demographic filtering, allowing users to restrict analyses to subsets, such as female patients over 60. Even within such filters, sample sizes remain large enough to detect meaningful differences, facilitating age- and sex-aware disease comparisons.

## Discussion

PBMCpedia enables robust, comparative immune profiling across diseases by offering a harmonized and openly accessible dataset with standardized preprocessing. By uniformly reprocessing raw data, applying stringent quality control, and integrating hierarchical cell type annotations, the atlas ensures reproducibility and comparability across studies. Its two-level annotation system serves both novice users and expert immunologists, providing flexibility for various analytical depths. Stratified analyses by sex and age, together with multimodal data where available (TCR/BCR, CITE-seq), further enhance its utility for exploring immune heterogeneity at multiple molecular levels.

PBMCpedia’s technical innovations lie in its multistudy harmonization, transparent QC metrics, and integrated multi-omic profiles, which collectively establish a platform for both discovery and diagnostic evaluation. The combination of consistent preprocessing, batch correction with Harmony, and hierarchical annotation addresses a key limitation of prior atlases. Moreover, the availability of standardized metadata and multimodal modalities supports integrative analyses of transcriptomic, clonotypic, and proteomic variation, enhancing both biological insight and methodological rigor.

To illustrate the diagnostic potential of the database, we applied a simple multilayer perceptron as a proof of concept for incremental training across studies. This experiment does not introduce a new annotation model; rather, it demonstrates how even basic classifiers can detect studies that systematically degrade performance, guiding preprocessing or reannotation and highlighting dataset consistency.

As a community-curated resource, PBMCpedia reflects the availability of public data. Some diseases, including cancers and rare conditions, remain underrepresented. Future versions will incorporate new studies as they become available, leveraging our reproducible pipeline to expand coverage. This includes especially the raw data of the Allen Immune, facilitated by the already shared annotation, and the “Inflammation Landscape” [2].

While transcriptomics is the core modality, PBMCpedia already includes some multimodal datasets. Future updates will expand this further, including spatial transcriptomics and cell–cell interaction networks. Despite robust batch correction, residual cohort differences may persist. We encourage users to leverage the provided metadata, especially age, sex, and control samples from multiple studies, for careful interpretation.

PBMCpedia addresses the growing need for reproducible, cross-disease immune profiling by enabling scalable, metadata-aware analysis and therefore supporting both discovery and hypothesis-driven research. All data, code, and results are freely available through an interactive web platform.

By prioritizing accessibility, consistency, and scalability, PBMCpedia serves as a valuable tool for immunologists, computational biologists, and clinical researchers. We invite community feedback and contributions to expand and refine PBMCpedia as an open resource for immune research.

## Conflict of interest

None declared.

## Supplementary Material

gkaf1245_Supplemental_File

## Data Availability

PBMCpedia is freely available at https://web.ccb.uni-saarland.de/pbmcpedia/.
