# Supplementary material for: PBMCpedia: a harmonized PBMC scRNA-seq database with unified mapping and enhanced celltype annotation
Source: Nucleic Acids Res. 2025 Nov 24;54(D1):D1216–21. doi: 10.1093/nar/gkaf1245 (PMC12807695; doi:10.1093/nar/gkaf1245)
Supplement: gkaf1245_Supplemental_File [file gkaf1245_supplemental_file.pdf]

Machine Learning Proof-of-Concept

Method

We tested model generalizability using incremental training of a simple Multi-layer Perceptron (MLP) classifier (scikit-learn v1.5.2) [18] across studies. For each of two annotation levels, we trained on sequential batches and evaluated on a held-out project (P09) using `partial_fit`. The same held-out project was used for all runs to ensure comparability, while the order of the remaining projects was randomly permuted in each iteration to assess robustness to training sequence. To evaluate performance, we calculated the weighted F1 score on the held-out dataset after each incremental training step. This metric accounts for both precision and recall across classes while weighting by class support. Accuracy, precision, recall, and F1 score were recorded after each update.

By averaging F1 scores across permutations, we could smooth out run-specific fluctuations and identify consistent patterns. This yielded a project-level performance ranking. While no specific "performance impactors" emerged that universally degraded results, the ranking highlights variation in how different studies contribute to overall model accuracy. Lower performance likely comes from higher variance or contain underrepresented or edge-case cell types that the model struggles to integrate. Tracking these projects across runs provides a way to flag studies that may require additional preprocessing, reannotation, or specialized handling in future training workflows.

Models were tuned across hyperparameters including `alpha` (0.0001; 0.001; 0.01), and loss function (constant; invscaling; adaptive) to optimze the weighted F1 score. No complex architectures were used; the model serves purely as a simple diagnostic to demonstrate proof-of-concept and highlight study-specific contributions to classification performance.

Results

We evaluated classifier generalization across diverse datasets using incremental training, where each batch corresponds to one study. The weighted F1-score was tracked throughout (Supplementary Figure 1A), with individual training runs in transparent gray and a smoothed average in red. Performance improves rapidly across the initial batches, reflecting consistent signal from early datasets. The model continues to refine its predictions as more heterogeneous data is added.

Notably, overall F1 scores vary between projects (Supplementary Figure 1B). This suggests that some projects introduce higher variance or contain edge-case cell types not well represented in earlier batches. Importantly, we cannot assume that the reference annotations are biologically perfect as some disagreements may reflect real biological ambiguity or annotation uncertainty rather than model error.

Rather than being a limitation, this behavior offers a valuable diagnostic window: the incremental setup allows us to identify where and why performance fluctuates, leading to better understanding of data quality and diversity. Importantly, despite local drops, the overall performance remains high and stable, highlighting that our training approach is robust to heterogeneity.

The final confusion matrix (Supplementary Figure 1C) highlights the overall classification performance, with high diagonal values for most major cell types. Monocytes (0.86), T cells (0.78), and erythrocytes (0.70) are classified with high confidence. As expected, overlap occurs among phenotypically similar or transcriptionally related populations, such as DCs, progenitors, and ILCs, where shared marker expression may blur class boundaries.

The diagnostic value of this analysis lies in showing that even a simple baseline model can be sensitive to the heterogeneity of included projects. Incremental training may reveal projects whose addition consistently lowered predictive performance, suggesting that such studies either contain rare or difficult-to-classify cell types, or have higher internal variance. Flagging these datasets provides a principled way to identify cases requiring additional curation or preprocessing. The experiment thus serves as a lightweight diagnostic framework for assessing database stability, rather than a new cell type classification method.

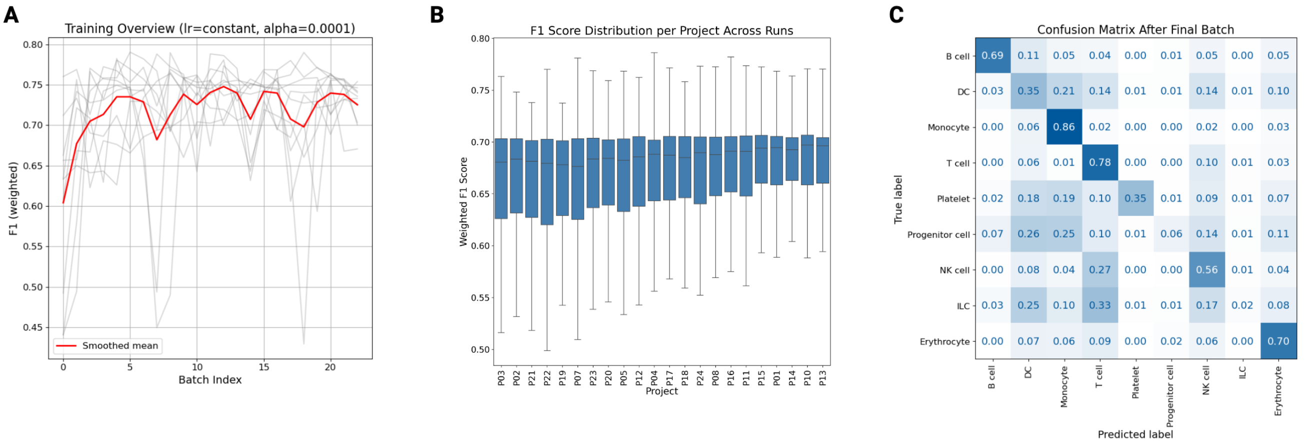

Supplementary Figure 1. (A) F1-score (weighted) across training batches. Each line represents a different training run with permuted batch/project order; the red line shows the smoothed mean. **(B)** Boxplot showing mean and standard deviation of the weighted F1 score for each project accross all runs. **(C)** Normalized confusion matrix after the final training batch, showing predicted versus true cell type labels.Created in BioRender. Hoffmann, E. (2025) https://BioRender.com/ lqe6s4z

ID	Study	Diseases	# Cells	Modalities
P01	Luo et al. (2022) [19]	-	139,111	GEX, TCR/BCR, surface protein
P02	Smith et al. (2020) [20]	-	9,620	GEX
P03	Yang et al. (2019) [21]	-	8,369	GEX
P04	Yazar et al. (2022) [22]	-	1,539,942	GEX
P05	Volden et al. (2022) [23]	-	3,131	GEX
P06	Goudot et al. (2017) [24]	-	850	GEX
P07	Xu and Jia (2021) [25]	Alzheimer's Disease	20,671	GEX
P08	Xiong et al. (2021) [26]	Alzheimer's Disease	74,109	GEX
P09	Bai et. al (2022) [27]	COVID-19	211.272	GEX
P10	Ivanova et al. (2023) [28]	COVID-19	507,223	GEX, TCR/BCR, surface protein
P11	Arunachalam et al. (2020) [29]	COVID-19	60,655	GEX
P12	Van der Wijst et al. (2021) [30]	COVID-19	25,848	GEX, surface protein
P13	Liu et al. (2021) [31]	COVID-19	1,047,036	GEX, TCR/BCR, surface protein
P14	Ramaswamy et al. (2021) [32]	COVID-19	252,643	GEX
P15	Unterman et al. (2022) [33]	COVID-19	95,819	GEX
P16	Lee et al. (2020) [34]	COVID-19, flu	96,992	GEX
P17	Zhang et al. (2023) [35]	End Stage Renal Disease	9,027	GEX
P18	Cillo. (2019) [36]	Head and Neck Squamous Cell Carcinoma	61,396	GEX
P19	Zhou et al. (2020) [37]	Inflammation	13,933	GEX
P20	Seyedsadr et al. (2023) [38]	Multiple Sclerosis	28,970	GEX
P21	Wang et al. (2022) [39]	Parkinson's Disease	27,427	GEX
P22	Zhang et al. (2023) [40]	Premature Ovarian Insufficiency	730	GEX
P23	Qiu et al. (2021) [41]	Sepsis	17,364	GEX
P24	Cai et al. (2020) [42]	Tuberculosis	41,055	GEX

Supplementary Table 1. Each row corresponds to one of the 24 publicly available single-cell PBMC studies integrated into PBMCpedia. For each study, we list the internal project ID used throughout the resource, the associated disease(s), the number of high-quality cells retained after preprocessing, and the modalities available. Modalities include gene expression (GEX), TCR/BCR repertoire profiling, and surface protein quantification (CITE-seq).

ID	# Cells	# Cells Removed	# Cells with > 10% MT [percentage]	Percentage of Cells with > 10% MT
P01	139,111	160,165	18,103	13.0%
P02	9,620	4,641	256	2.7%
P03	8,369	37,094	450	5.4%
P04	1,539,942	1,157,003	40,877	2.7%
P05	3,131	1,007	218	7.0%
P06	850	121	46	5.4%
P07	20,671	68,732	1,203	5.8%
P08	74,109	89,730	3,308	4.5%
P09	211.272	166,621	36,217	17.1%
P10	507,223	519,220	147,087	29.0%
P11	60,655	89,644	15,140	25.0%
P12	25,848	1,777	8,734	33.8%
P13	1,047,036	448,582	321.893	30.7%
P14	252,643	0	56.388	22.3%
P15	95,819	0	24,361	25.4%
P16	96,992	265,299	24,980	25.8%
P17	9,027	5,897	2,390	26.5%
P18	61,396	0	3,265	5.3%
P19	13,933	2,460	1,643	11.8%
P20	28,970	47,118	6,365	22.0%
P21	27,427	52,476	12,917	47.1%
P22	730	2,612	126	17.3%
P23	17,364	43,859	3,140	18.1%
P24	41,055	28,638	1,755	4.3%

Supplementary Table 2. Each row corresponds to one of the 24 publicly available single-cell PBMC studies integrated into PBMCpedia. For each study, we list the number of high-quality cells retained after preprocessing, and the number of cells exceeding the 10% mitochondrial threshold, as well as the percentage.
